# Can Cultured Meat Be an Alternative to Farm Animal Production for a Sustainable and Healthier Lifestyle?

**DOI:** 10.3389/fnut.2021.749298

**Published:** 2021-10-04

**Authors:** Camelia Munteanu, Vioara Mireşan, Camelia Răducu, Andrada Ihuţ, Paul Uiuiu, Daria Pop, Alexandra Neacşu, Mihai Cenariu, Ioan Groza

**Affiliations:** ^1^Department of Plant Culture, University of Agricultural Sciences and Veterinary Medicine Cluj-Napoca, Cluj-Napoca, Romania; ^2^Department of Fundamental Sciences, University of Agricultural Sciences and Veterinary Medicine Cluj-Napoca, Cluj-Napoca, Romania; ^3^Department of Technological Sciences, University of Agricultural Sciences and Veterinary Medicine Cluj-Napoca, Cluj-Napoca, Romania; ^4^Clinic of Obstetrics and Gynecology II “Dominic Stanca, ” University of Medicine and Pharmacy “Iuliu Hatieganu” Cluj-Napoca, Cluj-Napoca, Romania; ^5^Department of Chemical Engineering, Babeş-Bolyai University, Cluj-Napoca, Romania; ^6^Department of Animal Reproduction and Reproductive Pathology, University of Agricultural Sciences and Veterinary Medicine Cluj-Napoca, Cluj-Napoca, Romania

**Keywords:** cultured meat, health, greenhouse gases, bioreactor, animal welfare

## Abstract

Producing animal proteins requires large areas of agricultural land and is a major source of greenhouse gases. Cellular agriculture, especially cultured meat, could be a potential alternative for the environment and human health. It enables meat and other agricultural products to be grown from cells in a bioreactor without being taken from farm animals. This paper aims at an interdisciplinary review of literature focusing on potential benefits and risks associated with cultured meat. To achieve this goal, several international databases and governmental projects were thoroughly analyzed using keywords and phrases with specialty terms. This is a growing scientific domain, which has generated a series of debates regarding its potential effects. On the one hand the potential of beneficial effects is the reduction of agricultural land usage, pollution and the improvement of human health. Other authors question if cultured meat could be a sustainable alternative for reducing gas emissions. Interestingly, the energy used for cultured meat could be higher, due to the replacement of some biological functions, by technological processes. For potential effects to turn into results, a realistic understanding of the technology involved and more experimental studies are required.

## Introduction

According to the United Nations (1), the world population has grown over the past 10 years, reaching nearly 7.6 billion people in 2017, while it is estimated that by 2050 it will reach 9.8 billion people. Population growth, urbanization and income growth in developing countries along with the high importance of animal products in the majority of the global population's diet (2) have led to a search for sustainable alternatives to conventional products with less negative impact on the environment and, conversely, even on health.

**Figure 1:**
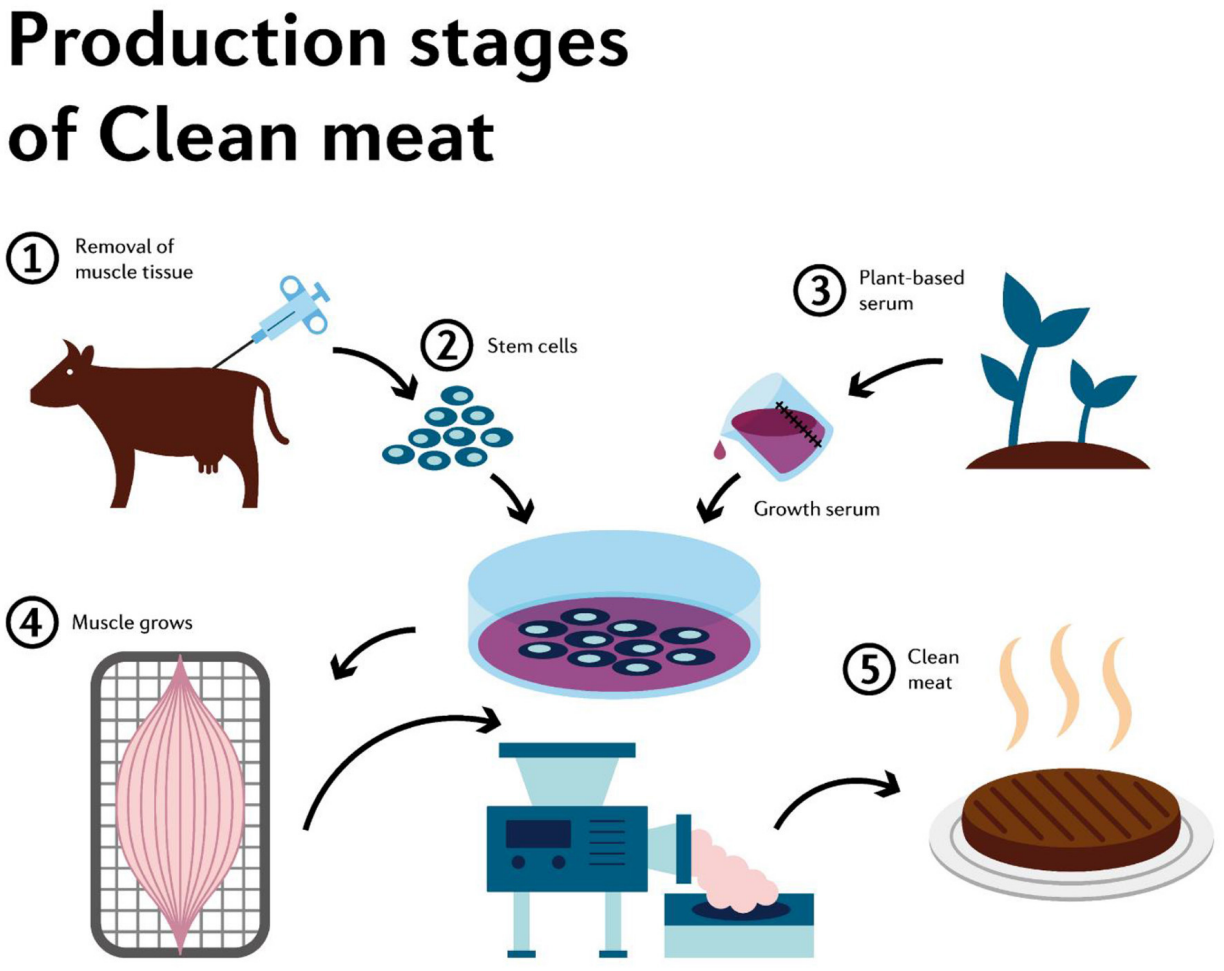
Culture meat cycle. Source: (9). 1- non-invasive uptake of stem cells from cows; 2 and 3 represent the steps in which cell culture takes place in a cultured environment to grow and divide; 4 - differentiation of cells tissues that are identical to the one harvested from the animal; 5-meat processing in order to reach the consumer directly.

Livestock breeding requires a significant amount of natural resources and plays a big role in global greenhouse gas emissions (3). Also, conventional meat production has other side effects, such as a decrease in nutritional value, foodborne illnesses and depletion of environmental resources, along with the suffering and slaughtering of animals (4). Post (5), believes that these alternatives should generally be based on sustainability, the reduction of environmental impact and increasing animal welfare. In addition, a 2010 UN Environment Program report showed that a significant reduction of agriculture's environmental impact would only be possible with a substantial change in the global diet (6, 7). From this perspective, cultured meat can be an alternative. According to Mattick (8), cellular agriculture is a revolutionary technology that makes it possible for both meat and other agricultural products to be grown from cells in a bioreactor without being taken from farm animals (Figure 1) (9).

This paper aims at an interdisciplinary review of literature focusing on the potential benefits and risks associated with cultured meat analyzed from both environmental and medical points of view. Interventional studies involving animals or humans, and other studies that require ethical approval, must list the authority that provided approval and the corresponding ethical approval code.

To achieve this goal, several international databases and government projects were thoroughly analyzed using keywords and phrases containing specialty terms such as “cellular agriculture,” “cultured meat,” “*in vitro* meat,” “health,” “gas emissions.”

In this paper, we have gathered and presented the results of several studies, reviews, reports and government projects. These studies cover a long period of time from 2000 to 2021. Thus, this review will emphasize on several aspects of cultured meat production, including the following issues: bioreactors (comparative analysis of the existing equipment and an original proposal), current status analysis of cultured meat bioprocessing, as well as advantages and disadvantages of cultured meat consumption.

## Bioreactors Used for Cultured Meat Production

Bioreactors are devices in which biological and/or biochemical processes develop under the precise control of environmental and operating conditions (pH, temperature, pressure, nutrient supply and waste disposal) (10). It all stems from the form and typology of the final product, as for example, processed meat vs. completely carved meat, or a source of dry protein in a powder form vs. a wet cell biomass. All this has a great impact on the type of bioreactor to be chosen. The purpose of a bioreactor is to generate an adequate controlled environment for the *in vitro* management of mammalian cells (11). The two sequential phases of the cell culture process, namely proliferation and differentiation form the basis of the bioprocess design, because the cultured cells are the desired final products. The design is iterative, as the choices must be made together with the calculations of mass balances, energy balances and methods of heat supply/disposal, including the integration of heat to save energy. An example of an upstream and downstream project iteration is used for recycling in order to save water and the size of waste recovery units to use waste products. All this is dependent on the flow rate of the bioreactor effluent. Along with the bioreactor for proliferation and differentiation, the upstream process probably includes units such as environmental storage tanks, environmental heat exchanger and a means of maintaining isothermal conditions (constant temperature) in the bioreactor. All these characteristics are presented in Table 1.

**Table 1:** The efficient characteristics of bioprocess in bioreactors.

Bioprocess: needs	Product formulation, upstream units, downstream units, pipelines, pumps, valves, heat exchangers, storage vessels, instrumentation e.g., temperature and pressure sensors, flowmeters, control system, capital cost-infrastructure, units, raw material sourcing, packaging, labeling
Cells	Type of cells used for culture Myoblast, myosatellite, iPSCs, etc. Primary-isolation, purification, verification Immortalized-cell line development Specific properties of cells Cell specific growth rate Method of inducing differentiation Anchorage dependent or non-adherent Metabolic stoichiometry Degree of differentiation required Size, mass (dry or/and wet), phenotype
Medium	Concentration and composition of essential nutrients, growth factors, vitamins. Rates of substrate consumption By-product production rates Source and variability e.g., serum, serum-free, chemically defined pH, osmolality, compound degradation Purchasing form: pre-formulated, powder, concentrates Storage requirements e.g., refrigeration impacts energy requirements In-house formulation e.g., culture grade water required and sterilization
Scaffolds	Cytocompatible-non-toxic, supports cell attachment, viability and proliferation/differentiation Cell dissociation method for passaging and/or end-of-batch processing (with or without porous) Material (natural or synthetic) Surface area to volume ratio Edible, biodegradable or non-degradable Final form e.g., microcarrier, hydrogel Source of final scaffold form: commercial supplier or in-house fabrication Mechanical and surface properties e.g., stiffness, striations Raw material source: sustainable, animal derived, etc.
Bioreactor	Type e.g., agitated with ball or perfusion (classically or direct) Mode of operation: batch, fed-batch, continuous (perfusion) Passaging method & requirements Inoculum method & density Homogenous environment – mixing, shear stress, sparging, heat supply/removal Monitoring & instrumentation: temperature, pH, dissolved oxygen, carbon dioxide, nutrients and byproducts, osmolality Cleaning and sterilization Proliferation/differentiation phase Oxygen supply Scale-up
The formulation of final product	Cell dissociation (if non-edible) or cell scaffold complex extraction Cell/tissue harvesting Product formulation Product unit size Packaging and labeling
Waste treatment and recycling	Waste identification e.g., by-products such as ammonia and lactate Waste separation e.g., electrodialysis, filtration By-product recovery Re-usable media recovery, recycle, additional substrate supplementation Waste valorization/up-scaling e.g., use as feedstock for alternative process/industry Energy and cost

In this respect, high reproducibility, control and automation of bioreactors for specific experimental bioprocesses are key to their transfer toward large-scale applications. In general, bioreactors are used in industrial fermentation processing, wastewater treatment, food processing and the production of recombinant pharmaceuticals and proteins.

As a field, tissue engineering has been defined as the application of engineering principles and methods in life sciences to the development of biological substituents to restore, maintain or improve tissue function (12). Normally, the most typical approach is to make 3D tissue structures generated by associating cells (autologous or allogeneic) with porous scaffolds, which provide the pattern for tissue development. They subsequently degrade or are reabsorbed at defined speeds.

The *in vitro* culture of 3D cell-scaffolds is performed under conditions that support efficient cell nutrition, possibly combined with the application of mechanical forces for direct cellular activity and with phenotype. It is thus a paramount step for the development of functional grafts in the treatment of lost or damaged parts of the body (13). Moreover, the designed tissues could provide reliable model systems, which allow a much better understanding of the structure-function relationships both under normal and pathological conditions. The result of these processes may have commercial applications in molecular therapy (e.g., drug screening) (14). In addition, the generation of 3D vivo tissues in addition to the development of new biological models (15) requires new technical challenges, due to the particular physico-chemical requirements of large cell masses. Cellular seeding of scaffolds – i.e., the dissemination of isolated cells in a scaffold - is the first step in establishing a 3D culture and could play a crucial role in determining the progression of tissue formation (16).

Indeed, high-density scaffold cell culture has been associated with improved tissue formation in 3D constructions, which includes higher rates of cartilage matrix production (17), increased bone mineralization (18), and improved heart tissue structure (19). Thus, the engineering of autologous grafts for clinical applications involves the use of high initial cell densities. However, while limiting the size of the biopsy and/or the degree of cell expansion, it is required that the cells be seeded as efficiently as possible. Moreover, the initial distribution of cells in the scaffold after seeding was related to the distribution of tissue subsequently formed in the designed constructs (20), suggesting that even cell seeding could establish the basis for even tissue generation.

The most efficient and even results were obtained when the cells were seeded in bioreactors equipped with stirring flask (21). Mixing the diluted cell suspension around the stationary scaffolds suspended from the mouth of the flask transports the cells into the scaffold by convection. However, most likely due to inefficient cell convection in the inner scaffold region, seeding in stirring flask bioreactors may also have low seeding efficiency (19, 20). In terms of cell distribution, it may become uneven (21, 22), with a higher density of cells lining the surface of the scaffold (23).

Applying the principle of convective transport for scaffold seeding, the flow of a cell suspension directly through 3D scaffold pores using a multi-pass filter seeding technique produced more evenly distributed cell scaffolds compared to static seeding (24). When direct infusion was incorporated into an automatic bioreactor for seeding 3D scaffolds, higher seeding efficiencies and more uniform cell distributions were obtained compared to static seeding or the stirring flask bioreactor (22). By using this simple concept, a variety of scaffolds can be seeded efficiently and reproducibly in an automatic and controlled process.

Interestingly, infusion seeding can be easily integrated into an infusion bioreactor system (Table 2) capable of performing both scaffold seeding and subsequent culture of the preparation. These seeding and culturing bioreactors were designed for vascular graft engineering (32) and were used in cartilage (33) and heart tissue engineering (34) and in maintaining hepatocyte function in 3D scaffolds (20).

**Table 2:** Bioreactors.

**Type of bioreactors**	**Advantages**	**Disadvantages**
Stirring flask bioreactors (Figure 2)	- The most efficient and even results obtained - Cells distributed more evenly compared to static seeding (26)	- Low seeding efficiency in the inner region of the scaffolding - The distribution of cells may become uneven, with a higher density of cells lining the surface of the scaffold
Perfusion bioreactors (Figure 3)	- Simplifies the engineering process, and reduces the safety risks associated with the handling and transfer of biological preparations between separate bioreactors. - Seeding the scaffolding, as well as the subsequent cultivation of the preparation - Vascular graft engineering (28) - Cartilage and heart tissue engineering and maintaining the function of hepatocytes in 3D scaffolding. - Simplifies the engineering process, and reduces the safety risks associated with the handling and transfer of biological preparations between separate bioreactors	
Direct perfusion bioreactors (Figure 4B)	- Improve: growth, differentiation and deposition of the mineralized matrix by bone cells (30), - proliferation of human oral keratinocytes, - rates of albumin synthesis by hepatocytes, - expression of specific cardiac markers by cardiomyocytes. - Substantially improves the survival, growth and function of all cells.	- The effects of direct infusion may be highly dependent on the average flow rate and the stage of maturation of the preparations (31) - The mechanism of applying mechanical forces to 3D constructions could be much improved

These systems not only simplify the engineering process, but also reduce the safety risks associated with the handling and transfer of biological preparations between separate bioreactors.

The most successful method is to use bioreactors that perfuse the environment through or around the semi-permeable hollow fibers of the scaffold, because the function of cells with an intense metabolism is maintained. This can be done by increasing the transport of nutrients and oxygen to the culture preparation. This concept has been extended to cultured tissues by perfusing the culture medium directly through the pores of the 3D scaffold with initial cells, which results in the reduction of mass transfer limitations. Direct perfusion bioreactors have been shown to improve: growth, differentiation, and deposition of mineralized matrix by bone cells (35), proliferation of human oral keratinocytes (36), rates of albumin synthesis by hepatocytes (20), the expression of specific cardiac markers by cardiomyocytes (37). Therefore, when direct perfusion is incorporated into a bioreactor, it substantially improves the survival, growth and function of all cells. It seems that even in this case, depending on the results of several studies for 3D cultures of chondrocytes, things are not perfect, because the effects of direct perfusion can be very dependent on the average flow and the stage of maturation of preparations (33). Therefore, the optimization of an infusion bioreactor for 3D tissue engineering must strike a careful balance between mass transfer of nutrients and residual products to and from cells, retention of newly synthesized extracellular matrix components within the construct, and shear stresses induced by fluid in the scaffold pores. Furthermore, the mechanism for the application of mechanical forces to 3D constructs could be much improved, beyond the conventional approach of improving cell differentiation and/or deposition of the extracellular matrix in the prepared tissues.

**Figure 2:**
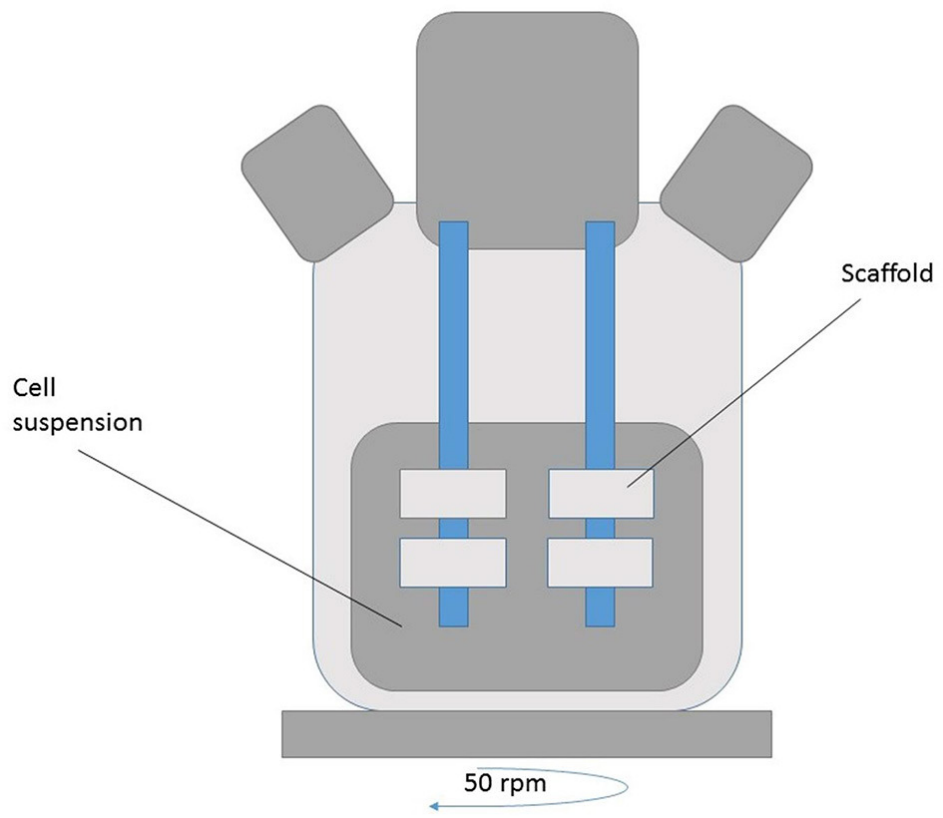
spinner flask bioreactor [adapted after (25)] is a type of stirred bioreactor which is formed by vessels that are responsible for gas exchange via side arms with loose screw caps. The mixing is realized by a non-suspended magnetic stir bar. Through two to four needles the scaffolds are fixed in a stopper in the mouth of the flask. Only after media inoculated cells filled the flask, all the compositions are stirred.

**Figure 3:**
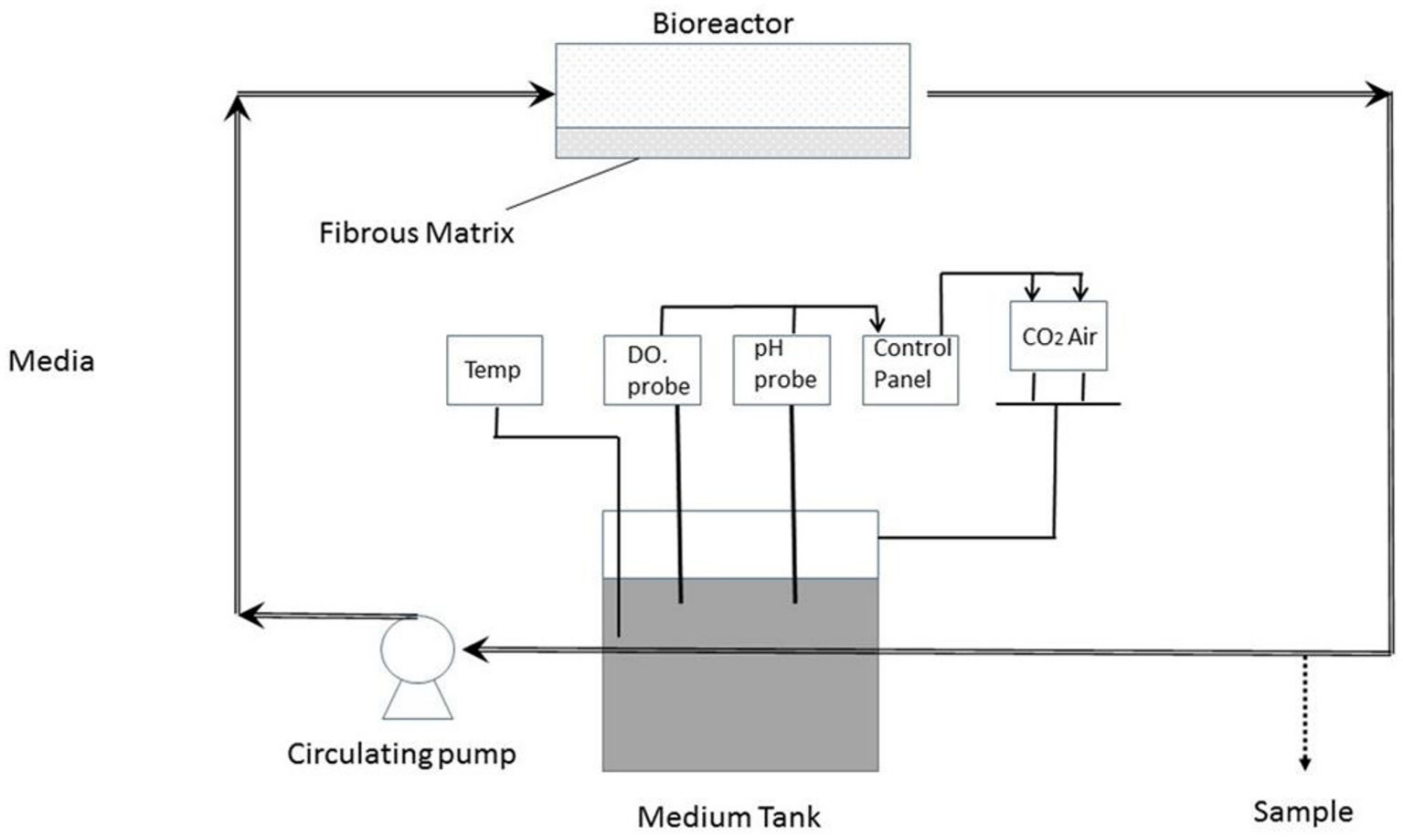
Perfusion bioreactor [adapted after (27)] - The cells are seeded into PET fibrous matrix and incubated for 1 day in a CO_2_ incubator; scaffolds have usually adherent cells; In the bioreactor chamber, the cells are placed horizontally; The culture medium is pumped in a parallel direction to cell-matrix through the chamber at a low flow rate; The temperature usually is at 37C; The pH is maintained at a physiological range of 7.2–7.4.

**Figure 4:**
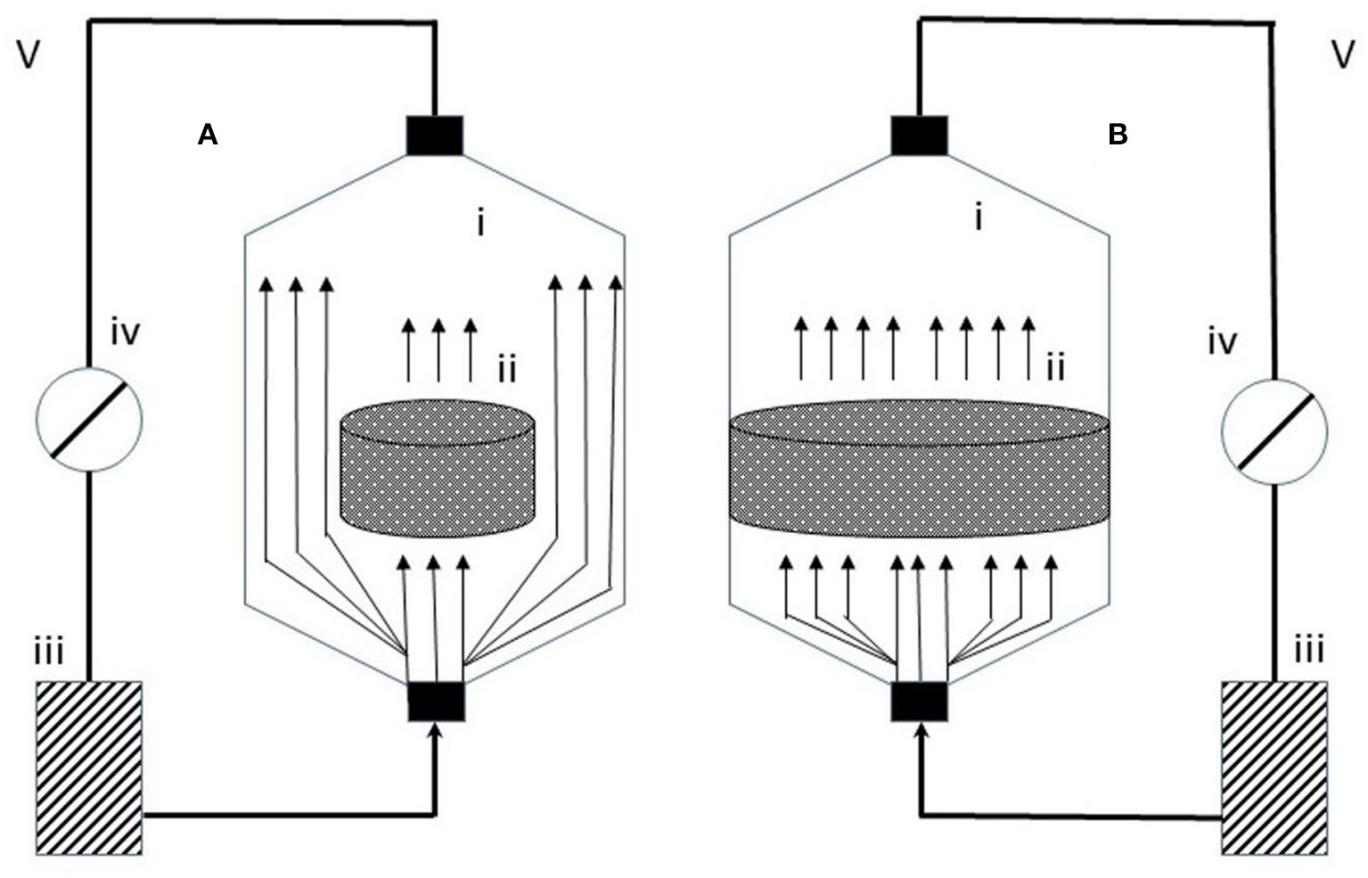
Indirect **(A)** and direct **(B)** perfusion bioreactor [adapted after (29)] - a simple representation of indirect and direct perfusion bioreactors which are made of the culture chambers (i), the cell/scaffold constructs (ii), the culture medium reservoirs (iii), the peristaltic pumps (iv), and the tubing systems (v); The culture medium from indirect perfusion bioreactors has a specific characteristic because it follows the path of less resistance around the constructs. In direct perfusion bioreactors, the cell/scaffold is in a press-fitted fashion in the culture chamber; The medium is perfused throughout the constructs.

**Figure 5:**
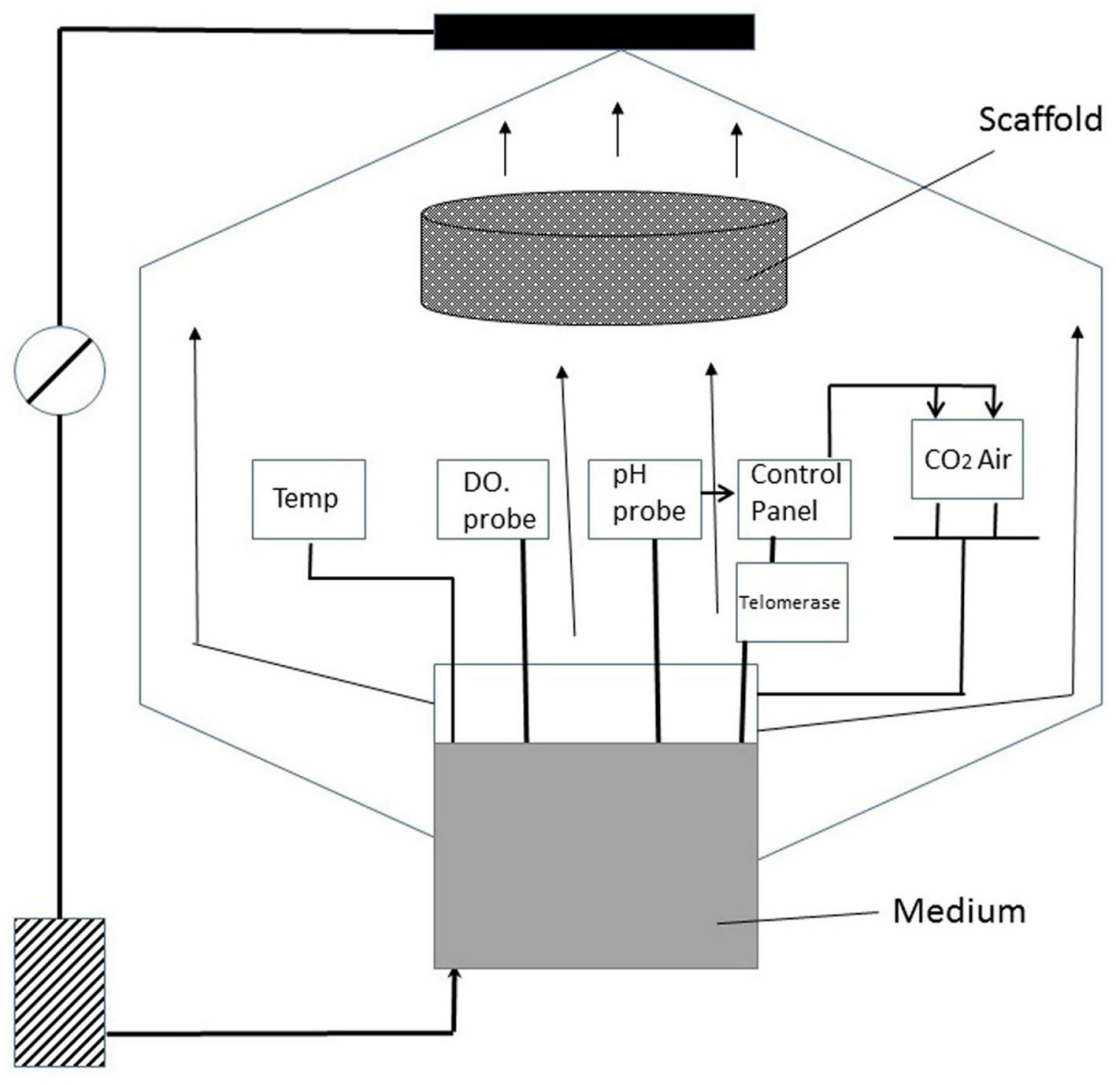
Our proposal of bioreactor (original) is similar to a perfusion bioreactor with a small difference; This change is represented by the adding of telomerase to decrease the Hayflick limit; Regulating the expression or exogenous addition of telomerase can effectively enhance the potential for cell regeneration, which is conducive to large-scale, stable, and rapid proliferation of animal cells.

They can serve as *in vitro* models to study the pathophysiological effects of physical forces on developing tissues. It could also predict the responses of newly formed tissue to physiological forces during surgical implantation. Moreover, bioreactors together with the mechanical response of the preparation could help to define the best time for the processed tissues to have sufficient mechanical integrity and a normal biological reaction in order for it to be implanted (Figures 2–5) (38).

Next, this review will present the bioprocesses of cultured meat, by analyzing the current status of this procedure and discussing the possibilities of improvement.

## Bioprocess of Cultured Meat

Nowadays, cultured meat can be obtained using several methods which include cell cultivation from muscle biopsies, tissue engineering as well as organ printing and nanotechnology (39, 40). Currently, these methods can only be applied at a small scale and are not yet adequate for large scale and commercial production (41, 42).

Certainly, a further question arises: how can the whole process be transferred onto a large, industrial scale? Furthermore, whether this process will not have negative effects on human health in the short and long term? These questions are closely related to the fact that growth hormone promoters are banned in agricultural systems for conventional meat production in the European Union (unlike other parts of the world).

Nevertheless, it is a long way to the real muscle, which consists of orderly fibers, blood vessels, nerves, connective tissue and fat cells (43). This is why different start-ups working in this field have developed different strategies: some of them work with stem cells or muscle cells to reproduce disorganized muscle fibers, which is the simplest approach, while others try to reproduce thin slices of muscle (i.e., muscle fibers and other cell types quite well-nested together).

However, producing a thick piece of meat similar to a real steak is still a dream, due to the need to infuse oxygen inside the meat to mimic the diffusion of oxygen as it appears in real tissue (44). Everything starts from stem cells that are collected in a non-invasive way from an animal. Subsequently, the cells are grown in a controlled environment, which provides favorable conditions for growth and division. Finally, it distinguishes itself into a tissue that is identical to the meat harvested from the animals (8). Two types of stem cells could be used: embryonic or myosatellite skeletal muscle cells.

When it comes to the cultivation of cultured meat, a typical material used for scaffolding is represented by the collagen spheres that transform stem cells into myotubes (45).

Stem cell technology for *in vitro* meat production was explored many years ago, but has not been industrialized yet. Nevertheless, there are some authors who believe that the development of stem cell and tissue engineering ensures the possibility of large-scale cultured meat production (46, 47). Cultivated meat requires a large number of differentiated muscle cells to form tissues. As it was pointed out before, maintaining healthy cells is achieved by providing fresh nutrients, while increasing the number of cells by maintaining constant cell division (48).

However, most cells have a limited ability to divide, known as the Hayflick limit, which restricts muscle tissue cultures in a large-scale laboratory. In this regard, effective ways have been developed to increase proliferation, including increasing the regenerative potential of stem cells. It is known that the Hayflick limit is determined by the length of the telomeres, which is a repeated sequence at the end of the guanine-rich chromosomes. Telomeres shorten with each replication, affecting the cell's ability to proliferate. Telomerase, which prolongs telomeres, is found in anti-aging cell lines. Therefore, regulating the expression or exogenous addition of telomerase can effectively enhance the potential for cell regeneration, which is conducive to large-scale, stable, and rapid proliferation of animal cells (49).

In addition, another major challenge is the need to support high cell densities to match those in native tissue (108–109 cells/ml) (50). However, most research on cultured muscle tissue has been performed at relatively low cell densities (107 cells/ml) (51). Adapting to the need for high encapsulated cell density may require adapting scaffolding to ensure manufacturing feasibility and optimize the cellular microenvironment. Moreover, muscles are made up of muscle fibers with different metabolic rates. The question is how is it possible to maintain this difference *in vitro* to copy the native tissue as accurately as possible? Alternative approaches to maximize protein content in cultured meat prototypes include the use of edible, protein, or biodegradable scaffolds that are replaced by cells and their ECM proteins secreted during culture (52).

**Figure 6:**
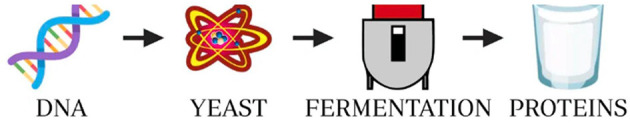
Meat substitutes-acellular food production. Source: (57) First of all, acellular food production starts with a DNA sequence; Then it is inserted into yeast and makes fermentation, subsequently yielding new cells; The new cells are genetically identical to the mother cells.

Historically, the NASA institute played an important role in the history of clean meat technology: it pioneered the production of muscle protein substitutes for astronauts and space station inhabitants. Over time, different approaches to potential *in vitro* meat production began to emerge (4). All this culminated with the creation of the first meat burger in 2013, cultivated by Professor Mark Post from the University of Maastricht, at a conference in London 2013 (53). Collaboration projects have started to appear in recent years among companies such as Mark Post's Mosa Meat (Netherlands), Aleph Farms (Israel) and Cellular Agriculture Ltd (United Kingdom) and some prestigious universities, which carry out biomedical academic research programs on this topic (54). Therefore, most of the research in the field is conducted within start-up companies, which can be selective regarding the information provided to the public about the technological process. Thus, it is quite difficult to know precisely what each company does (55). In parallel, meat substitutes were created and developed. They are entirely plant-based. Most products contain soy, milk protein, whey protein or mushrooms, while also meeting all criteria for efficient low carbon footprint protein production (56). It must be pointed out that there is also cellular agriculture based on fermentation and it is different from the one based on tissue engineering, as it does not use tissues from living animals. Genetically modified bacteria, algae or yeasts were used to obtain fermentation-based products by adding recombinant DNA to produce organic molecules (Figure 6) (57).

As such, obtaining some known animal products (gelatin, casein and collagen) is based on the use of these molecules (Clara Foods - egg white, Perfect Day - formerly Muufri - milk). This type of agriculture is different from cellular tissue engineering because the products related to this technology can be traded in a shorter period and are based on the usual industrial biotechnology. Also, the technology used in cellular tissue engineering agriculture has not been proven efficient for large-scale production (58).

Most of the time, people have been skeptical and even dismissive when confronted with advanced technologies, especially when it comes to food. Therefore, this topic has generated a series of debates regarding the possible beneficial effects and the risks for the environment and health. According to Hocquette et al. (58), *in vitro* meat can reduce the carbon footprint and meet all the nutritional needs and desires of the consumers. Some researchers even suggest that cell agriculture is going to have beneficial effects, starting from increasing animal welfare to improving human health (10). Van der Weele and Driessen (59) claim that cultured meat can be a healthier alternative because it is safer to control the bacteria and viruses in cell cultures than in animals, while they also claim that cultured meat could be enriched with healthy compounds. There are also supporters of “non-animal products,” that ask themselves what meat, milk, eggs and other products obtained from cellular agriculture are (6). Interestingly, Mattick (8) has another vision on this technology and shows that when it comes to the production of pork and poultry, greenhouse gas emissions may be twice as high as those produced using conventional techniques, because the consumption of energy associated with *in vitro* meat is much higher.

**Figure 7:**
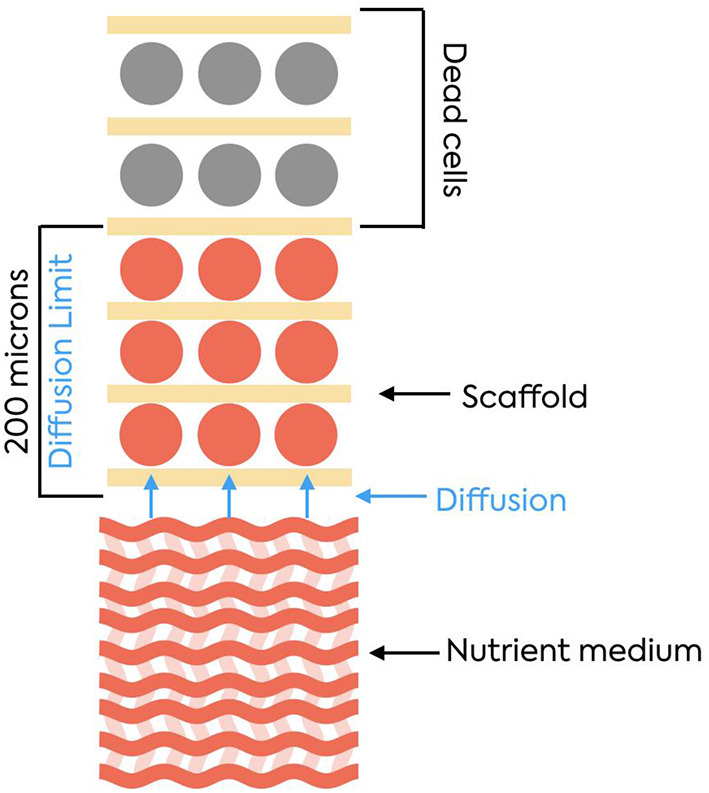
Extracellular matrix. Source: (60) represents a structure that contains macromolecules, like collagens, enzymes, and glycoproteins; These help the cells grow by providing mechanical support; ECMs also have complex signaling molecules so the cells can communicate with one another.

In addition to bioreactors, stem cells and medium, artificial scaffolds (Figure 7) (60) and scaffold-based tissue engineering strategies are needed to obtain cultured meat. The natural question is “what are artificial scaffolds?” They represent the equivalent of the extracellular matrix. *In vitro* meat production for processed meat products requires the large-scale cultivation of stem cells in large bioreactors. Stem cells and skeletal muscle cells need both a solid surface for cultivation and a large surface to generate the wanted amount of muscle cells. They resemble the architecture of the native tissue and provide a medium that allows the growth of culture volumes (45). There are 2 major types of bioreactors used in this industry: reusable ones (stainless steel) and single-use ones. They are chosen based on the specific process that is going to take place.

Cultured meat production using the above-mentioned bioprocesses has obvious advantages regarding the impacts on environment, human health, land usage, water pollution and animal welfare. In the same time, it has some disadvantages referring to its quality, flavor and content. The following sections will show the benefits and drawbacks of cultured meat production and consumption.

## Advantages/Disadvantages of Cultured Meat

### The Impact of Cultured Meat on the Environment

This paper focuses on providing a comprehensive perspective on the impact of cultured meat on the environment and human health. For instance, the impact of cultured meat on gas emissions: given that environmental pollution is increasing and global warming is a real threat, scientists suggest a sustainable solution to stop the industrialization processes that increases greenhouse gas emissions. Livestock farming is responsible for the production of the highest percentage of greenhouse gases in the atmosphere, especially methane and nitrogen oxide, and consequently for increasing the carbon footprint. This argument is very relevant in terms of herbivores, which produce in their rumen a lot of methane (greenhouse gas), due to their digestive process (61). Methane affects global warming 28 times more than carbon dioxide. Furthermore, nitrogen oxide from manure storage and fertilizer usage has a global warming potential 265 times higher than carbon dioxide (3). Tuomisto and Mattos (62) showed that cultivated meat production substantially reduces GHG emissions. The carbon footprint depends on the variation in the quantity and composition of emissions associated with contemporary *in vitro* meat production systems. CH_4_ and N_2_O emissions are relatively small and therefore do not significantly contribute to global warming dynamics. In the case of CO_2_, the continuous emission rate determines the long-term increase in heating (63).

As a result, cultured meat has a clear advantage over classical meat production, making it possible to control the working environment and the disposal of ancillary products.

United Nations Sustainable Developments Goals (SDGs) of the 2030 Agenda mention Climate Action as one of the important strategies to be considered. Production of cultured meat could significantly reduce gas emissions and therefore contribute to the decrease of green-house effects (64).

Greenhouse gas emissions such as CO, CO_2_, NOx, CH_4_, etc. can be captured using specially designed filters and devices (65). Furthermore, the energy used for the production of grown meat is lower compared to the one used for conventional beef and sheep, but higher compared to the energy used for swine and poultry (8). These data are adequate for production equipment that uses a mixture of fuels of 43% natural gas, 33% coal and 16% electricity, similar to the one used for obtaining malt beverages (66). It is somewhat normal because all other organs/tissues that can't be found in cultured meat production play an important role in ensuring the vital functions of the body, circulation, respiration, digestion and excretion. When it comes to cultivated meat, all these functions must be performed using energy.

Therefore, everything depends on the type of bioreactor used. According to Edelman et al. (67), on a large scale, the production of cultured meat requires the design of new bioreactors to maintain low shear as well as a large, uniformly infused volume. There are research studies of skeletal muscle tissue engineering that used NASA rotating bioreactors. The benefit of these bioreactors is that the cells are in almost continuous suspension, the shear of the fluid is minimal, and the suspension is possible for tissue assembly that is up to 1 cm (11). As a result, on a small scale, the biological notions of cultured meat production are relatively well-understood and developed, but the technology of large-scale production of cultured meat remains at an early stage. In this regard, changes have occurred over time with cell source, scaffold, culture media and bioprocessing (11, 55, 67). In addition, one problem that is possibly associated with cultured meat is muscle atrophy, namely muscular loss due to cell size reduction (68) caused by lack of use, denervation or a variety of diseases (69, 70). In order to fix this problem, Edelman et al. (67) proposed a mechanical extension of the scaffold and of the extendable pearls for the scaffold in order to meet the requirements for ensuring contraction. Moreover, according to De Deyne (71), electrical stimulation can be performed on a large scale in order to induce internal contraction and serves to differentiate and form sarcomeres. Therefore, there are solutions to this possible problem.

### Impact of Cultured Meat on the Land Usage and Climate Change

The surface of the land and the amount of water used for this are much smaller compared to those used for conventional meat (62). Thus, 70% of all arable land on the planet represents the entire area required for animal husbandry (grazing land and feed production) (72). Production of cultured meat could reduce the need for arable land, thus natural habitats in various parts of the world could be restored, as stated by the United Nations Sustainable Developments Goals (SDGs) of the 2030 Agenda (Life on Land objective) (64).

### Impact of Cultured Meat on Water Pollution

Through cultured meat production, both water usage and water pollution are considerably decreased compared to what conventional agriculture produces (62), because the zootechnical section uses water for feed production, animal husbandry and sewage. About 33% of the global pollution with nitrogen and phosphorus is due to the water used for stables. Moreover, this water represents half of the antibiotic pollution and contains more than 30% of the toxic heavy metals that contaminate fresh water (72).

### Impact of Cultured Meat on Animal Welfare

**Figure 8:**
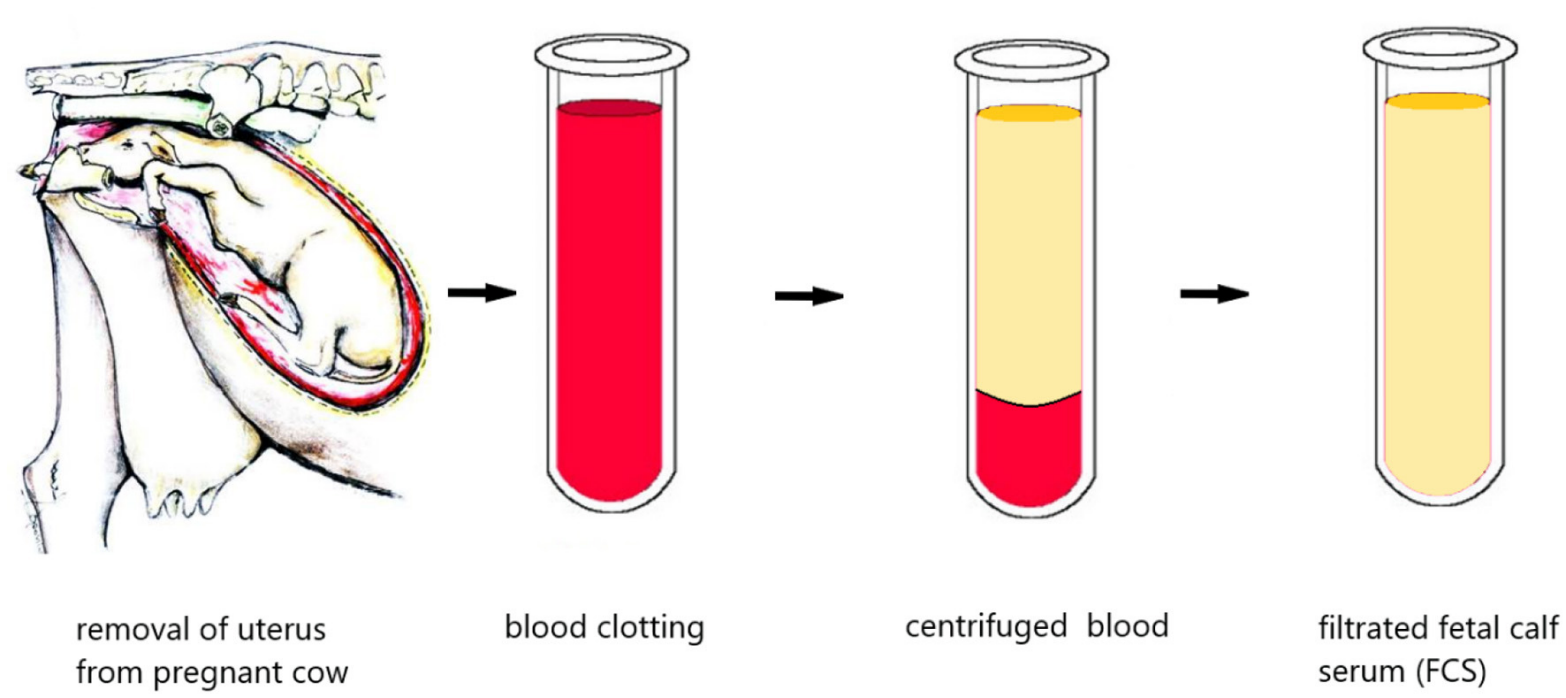
The obtaining of fetal bovine serum (original) this is taken by sacrificing a pregnant cow and draining the blood from the fetus's heart, which obviously has not been anesthetized.

In the scientific world, consensus has been reached on the health of animals and their ability to suffer, which is officially to be found in the EU animal welfare legislation (73, 74). Studies show that conscious experience is possible not only in the human brain, but also in animals (75, 76). Therefore, it is advisable to stop the suffering of animals under human care. Even if the EU has guidelines for ensuring animal welfare, it is specified that thorough animal health inspections should take place at proper intervals in order to avoid unnecessary suffering and in the case of animals raised on factory farms, at least once a day. However, these rules are often disregarded. In addition, the suffering caused by collecting stem cells from animals is negligible compared to that of animals in the conventional meat industry. Stem cell collection is performed under local or complete anesthesia, it does not take more than a few minutes and involves the use of a biopsy syringe and presents a low risk of long-term complications (77). According to Hopkins and Dacey (78) *in vitro* meat has the potential to reduce animal suffering considerably. Interestingly, the culture medium for cultured meat is represented by a sterile liquid that contains both essential macronutrients (sugars, amino acids) and micro-nutrients (vitamins, minerals). Currently, fetal bovine serum (FBS) is a key component of the standard culture medium used in biotechnology laboratories worldwide (79). Unfortunately, this is taken by sacrificing a pregnant cow and draining the blood from the fetus's heart, which obviously has not been anesthetized (Figure 8). This inhuman process is a major ethical issue against cultured meat (80).

Many researchers believe that ideal culture media should be free of nutrients from animals (80). In addition, culture media based on plants, fungus and microalgae have already been proven to work, which is why they should be used exclusively.

### Impact of Cultured Meat on Human Health

Cultured meat can have beneficial effects on health, because it ensures control over the composition and quality of the meat. It can change the flavor, fat content and, in particular, the ratio between saturated and unsaturated fatty acids (11). Based on the fact that culture meat only includes muscle, it will not contain hormones and microorganisms that can endanger human life (such as Salmonella). Hormones are usually used to accelerate the growth of animals in order to increase the production of meat and seafood. Also, *in vitro* meat will be free of dioxins, antibiotics etc., which are usually found in conventional meat (4). Cell agriculture may lower the incidence of obesity and cardiovascular disease and significantly reduce the number of cases with foodborne illnesses, as well as reduce the incidence of animal-transmitted diseases (8, 11). Edwards (81) reported that 60% of human diseases and 75% of emerging human diseases come from animal transmission. Thus, in humans, swine and avian influenza are transmitted from animals (82). Botulism becomes also a preventable disease by using controlled cellular agriculture.

**Figure 9:**
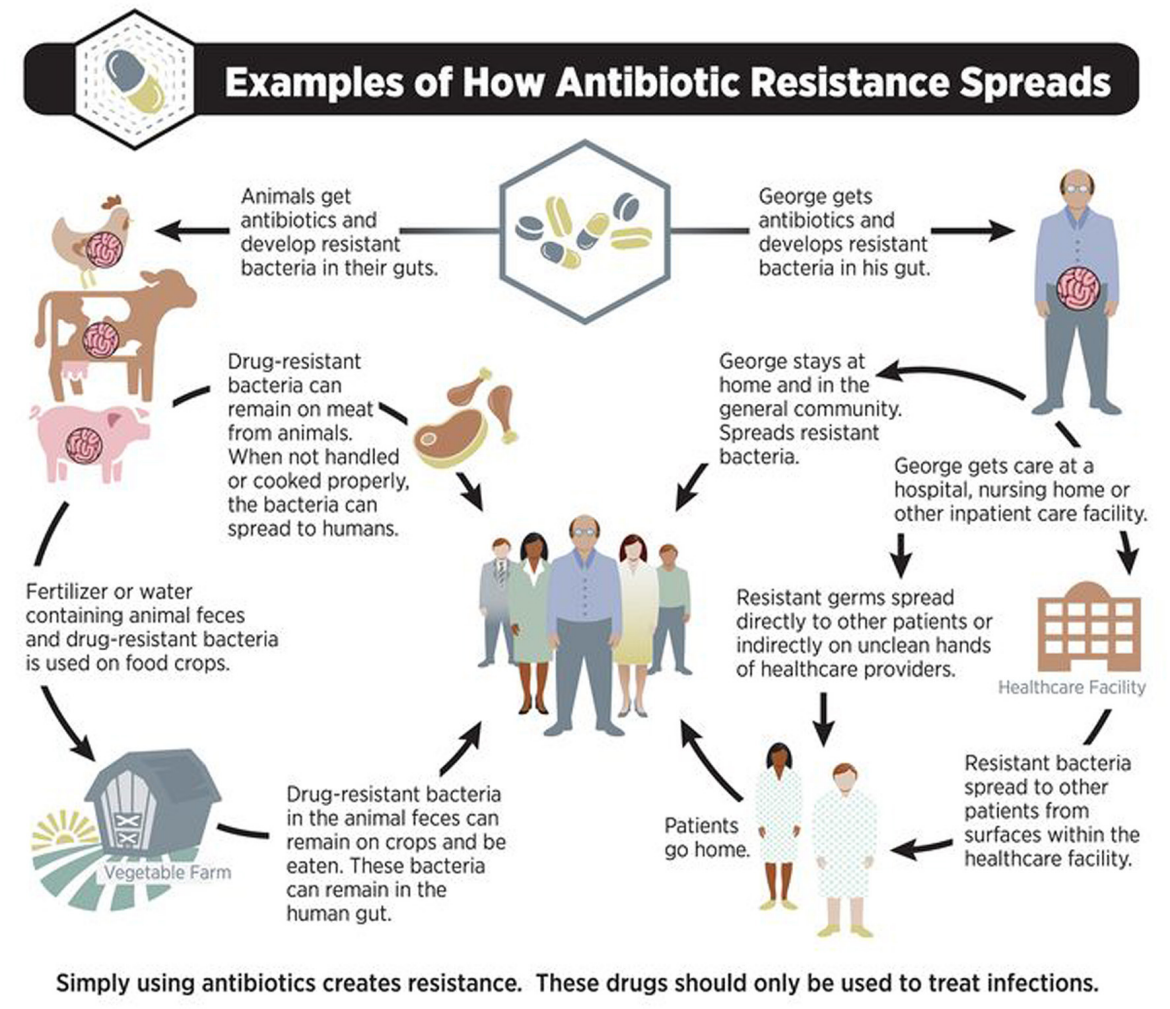
Antibiotic Resistance Spreads. Source: (86). The large use of antibiotics represents the most important factor leading to world antibiotic resistance; For prevention, control, and treatment of disease, and also to promote growth they are commonly used in food-producing animals; For the treatment of plant crops against bacterial infections antibiotics are used in very small amounts.

It has been reported by cultured meat producers that antibiotics are not used during the process, as opposed to conventional agriculture, where antibiotics are widely used in sub-therapeutic doses to help animal tissue growth. The widespread use of antibiotics in animals has led to human growth of significant pathogen-resistant strains (83). The World Health Organization considers antibiotic resistance to be one of the biggest threats to global health (84). Therefore, the concept of One Health has lately emerged, which refers to the link between environmental, animal and human health. Production and consumption of cultured meat could represent a feasible solution that would allow avoiding the transmission of antibiotic resistance in this life circle. Classical meat production sometimes involves the use of various antibiotic substances. They may be administered in an inappropriate way, considering the dose, duration of treatment and complying with the specific withdrawal period of each antimicrobial. Therefore, antibiotic residues are often found in meat from slaughtered animals and are consumed by humans, leading to allergic reactions as well as acquisition of resistance. Researchers from Michigan State University claim that due to the high number of factors that contribute to its development, antibiotic resistance is a polarizing topic that has led to numerous disputes between the scientific and industrial worlds (85). Such experts believe that one priority should be raising awareness and ultimately finding alternative strategies (Figure 9) (86).

However, what happens in the case of senescence? *In vitro* meat production systems that use satellite cells still bring up questions about senescence. These can be resolved either by replacing them with fresh cells whenever needed, or by immortalizing the cell culture, which is achieved most easily by using embryonic stem cell cultures. As for fresh satellite cells, they can be extracted non-invasively, to start new cell cultures (70). The latter solution involves genetic changes of the cultured cells in such a way that senescence can be overcome by altering the ectopic expression of the telomerase enzyme gene (87). This method, which pertains to the field of genetic modification, could make consumers refuse them. The last possibility involves embryonic stem cells that are pluripotent and, apparently, have a limited ability to divide (88), therefore, the culture of embryonic stem cells derived from a single donor can be used infinitely in theory. However, embryonic stem cells need to differentiate into muscle cells before they can be used.

In 2015, Consumer Reports conducted a study that showed that out of approximately 500 kilograms of beef bought by chance in several cities in the United States, 100% contained bacteria with fecal contamination. As for cellular agriculture, which has no meat from livestock (where the highest contamination occurs), there is no risk of fecal contamination (89). Even though cultured meat may have a completely different risk profile from conventional meat, special attention should be paid to the safety of the added substrates and other compounds of the culture medium.

Therefore, there are fewer risks for microbial contamination, but more risks for substrate contamination (11). For individuals with sciatica or other iron deficiency diseases, who choose to be vegetarian/vegan, cultured meat can be an alternative to cure iron deficiency (90). Also, compared to conventional meat production, cultured meat is devoid of hormones. Such hormones are used to accelerate the growth of animals in order to increase the production of meat and seafood. Also, as it is known, about eight million tons of plastic materials get into the ocean every year (91). Given that microplastics are linked to chemicals used in manufacturing, more and more reports on their physical and chemical toxicity have started to appear as they are ingested by many species of fish and shellfish. *In vitro* meat can reduce the pollution and toxicity caused by them. Also, by using cellular agriculture it is possible to avoid the contamination with residues in vegetable farms, too, when these are close to animal farms.

Another important issue regarding the use of cultured meat refers to the compliance with the United Nations Sustainable Developments Goals (SDGs) of the 2030 Agenda. The production and consumption of cultured meat could represent a sustainable solution to decrease of poverty and hunger. It is well-known that third world countries have an acute need for animal protein which could be replaced by cultured meat, if only it could be produced on a large scale and low cost. Also, good health and well-being could be improved by the consumption of cultured meat. It represents a healthier and non-contaminated source of nutrients, which can even be enriched by adding various other micronutrients which are missing from the diet in some geographical areas (64).

### Impact of Cultured Meat During Global Pandemic

Government measures on travel restrictions have created a scarcity in various economic sectors, including agriculture (92). However, with various restrictions and growing health problems, the demand for organic and local foods has increased significantly. Regional and local agricultural products are more sold compared to other products (93).

Although it is not known that SARS-CoV-2 can be transmitted through food and this has been explicitly emphasized by authorities such as the European Food Safety Authority (94), a decrease of meat consumption was reported during the pandemic compared to other years. Moreover, according to the United Nations Biennial Report on the World Food Outlook, meat consumption per capita in 2020 recorded the highest decline since 2000. According to Steinberg et al. (95), this may be the result of market disruptions caused by outbreaks of infection among workers in the meat industry and related closures or reduced operation of meat processing facilities. In addition, another factor that contributes to this phenomenon was generated by the closure of schools and restaurants imposed in response to the COVID-19 pandemic (96). Moreover, it is possible that the hypothesis that meat production and consumption increase health risks in terms of infectious diseases may be responsible for the dietary choices of some individuals (97). However, there are studies that have reported a general increase, lack of a significant decrease in meat consumption during nationwide blockages imposed in a number of countries (98).

### The Quality of Cultured Meat

The taste of real meat is influenced by the post mortem processing conditions, such as storage conditions, and pre-slaughter animal conditioning designed to preserve desirable meat quality. It is important to mention the fact that the metabolic transformation after death is represented by tissue hypoxia. The result is a decrease in intracellular pH (99). Moreover, acidic pH is responsible for losing water-binding capacity. As we expected, there are no studies regarding the quality of post-mortem metabolism of cultured meat, and basic studies to assess this process compare to animal-based meat are required given its significant contribution to meat properties (100).

Moreover, we are left with some questions, namely the nutrition value of cultured meat, because the taste, the texture is related to the nutritional specificity of the animals. In addition, if the animal has more intense physical activity, the meat is much richer in protein and low in fat. Also, the distribution of the type I or type II muscle fibers depends on the physical activity that certain subjects perform. This transition can be produced in the presence of B3 Vitamin (101). Probably in the function of the requirements, cultured meat producers have to adjust their products. Obviously, as is already known between these fibers, there are a number of metabolic differences, including those related to the metabolism of iron. The question is how culture meat can mimic these characteristics? Moreover, there are some compounds that are synthesized by endogenous cells but need exogenous supplementation. For example, as we have known the fats are responsible for the enhancer of good flavor for meats. Through oxidation, fatty acids can produce carbonyl compounds that are potent flavor contributors. Volatile compounds released from fat may be responsible for the species-specific flavors of beef, pork, and lamb (102). So, from this point of view, some fatty acids such as linoleic acid (103) and docosahexaenoic acid are synthesized through specific biohydrogenation from the ruminant gut or algae, not by cultured adipocytes (104). In addition, there are some vitamins like B12 (105) that are microbial-derived. Choosing a healthier diet which nowadays represents a dominant trend around the consumers is vital for meats producers to introduce healthy acids in their meat (106).

## Future Trends

Conventional agriculture is linked to several problems, such as the environmental ones, due to the increase of greenhouse gas emission, pollution of the land surface used in industrialization, with a hint toward carbon footprint and global warming. From this point of view, we propose the calculation of the bioprocess efficiency and according to its adaptation in order to obtain a large-scale production with the lowest possible costs and a minimum degree of pollution. The organoleptic properties of cultured meat should perfectly mimic conventional meat in order to reach the consumer directly.

Animal welfare issues need to be taken seriously. Certain aspects related to the impact of this type of agriculture on human health should not be neglected, such as the source of infectious diseases, antibiotics resistance, products with low nutritional value. Also, we recommend improving the culture meat with micronutrients to avoid some pathologies.

In this context, alternative solutions should be found, analyzed and verified, in order to find out if they are sustainable. Cultured meat may represent such an alternative, but as it was mentioned, it is in its infancy and for the time being, it cannot be performed on a large scale. For potential effects to turn into results, a realistic understanding of the technology involved and more experimental studies are required.

Future studies should be performed taking into consideration the use of novel bioreactor types. New technical solutions should represent an objective of such studies in order to facilitate large scale-production of cultured meat. Moreover, the effect of cultured meat consumption on human health should be assessed, as well as antibiotic resistance, correlated with the One Health objective. Another important goal is to study the impact of cultured meat production on the environment and the feasibility of such production from an ecological perspective. These proposals must comply with the United Nation's Development Goals (SDGs) of the 2030 agenda. Out of the 17 objectives proposed by this strategy, cultured meat production could contribute and bring some benefits to the following: zero hunger, good health and well-being, climate action and life on land (64).

## Author Contributions

CM, IG, and MC wrote the original draft and contributed substantially to the design of the research, to the collection, and interpretation of the data. VM, AI, CR, and PU determined a critical revision of the article and data curation. DP and AN contributed to the realization of the last version of the manuscript through conceptualization and investigation. All authors have read and agreed to the published version of the manuscript.

## Conflict of Interest

The authors declare that the research was conducted in the absence of any commercial or financial relationships that could be construed as a potential conflict of interest.

## Publisher's Note

All claims expressed in this article are solely those of the authors and do not necessarily represent those of their affiliated organizations, or those of the publisher, the editors and the reviewers. Any product that may be evaluated in this article, or claim that may be made by its manufacturer, is not guaranteed or endorsed by the publisher.
